# Experimental and numerical study on the effect of web openings on the torsional behavior of pre-compressed hollow UHPC beams

**DOI:** 10.1038/s41598-025-17420-4

**Published:** 2025-09-01

**Authors:** Ahmed M. El-Basiouny, Hamed S. Askar, Mohamed E. El-Zoughiby

**Affiliations:** Structural Engineering Department, Faculty of Engineering, Mansoura University, Mansoura, Egypt

**Keywords:** Ultra-high-performance concrete, Openings, Precompression, Hollow beams, Torsional behavior, Finite element method, Engineering, Civil engineering

## Abstract

Hollow ultra-high-performance concrete (*UHPC*) members subjected to axial pre-compression and torsion represent realistic loading scenarios commonly observed in modern engineering structures, including bridge box girders, prestressed members, and high-rise tubular columns. The inclusion of web openings further reflects practical design requirements. However, the combined effect of pre-compression, torsion, and openings on *UHPC* members remains insufficiently addressed in literature. To address this gap, the present study integrates experimental work with numerical simulation to provide novel insights into the structural behavior of *UHPC* beams under complex loading scenarios. The experimental study involves testing five *UHPC* pre-compressed reinforced hollow beams with central openings under torsion. Their results are presented in terms of cracking and ultimate torque, failure modes, cracking pattern, elastic and cracked torsional stiffness, post-cracking load-carrying capacity, torsional ductility, strain in lower steel bars and torque–angle of twist curve. In the numerical study, 23 *UHPC* beams (including the 5 tested beams) are modeled using the finite element method with Abaqus software. The presentation of numerical results includes some measurements that could not be experimentally reported. Based on the findings, key recommendations are also proposed to guide future design and implementation of *UHPC* members under combined loading.

## Introduction

Ultra-high-performance concrete (*UHPC*) offers the potential to become a practical solution to improve sustainability of buildings and other infrastructure components^1–4^. It exhibits superior mechanical properties, such as compressive strength (≥ 120 MPa under standard curing and 150 MPa under thermal curing^5^), high tensile strength, exceptional ductility (about 300 times that of high strength concrete (*HSC*)^6^), extraordinary durability and good flowability^5^. Furthermore, incorporating fibers improves its impact and toughness resistance^7^ and develops strain-hardening after cracking^8,9^. These characteristics, among others, allowed incredible levels of quality that had never been thought possible before, especially in bridges construction^6,10^. Zhou Mi et al.^11^ presented astonishing variable shapes of bridges, all over the world, that partially or totally designed using *UHPC*.

In curved bridges, as torsional stresses are predominant^12^, *UHPC* represents a suitable and practical choice to effectively resist such stresses. Also, precompression can be considered an appropriate solution to strengthen these bridges against the torsional stresses.

Recently, particularly in bridges, the need for creating openings in beams and girders has frequently increased; facilitating passing ducts and pipes for mechanical and electrical services, installing the connections between adjacent beams, enabling maintenance process, reducing the weight of concrete elements in long spans, preventing the buildup of harmful gases in enclosed environments and creating unique architecture designs for aesthetic appeal.

Owning to its superior mechanical properties, *UHPC* has attracted considerable research interest for strengthening normal strength concrete (*NSC*). Wissam Nadir et al.^13^ investigated the behavior of *NSC* beams strengthened in shear with FRP-Reinforced *UHPC* overlays, while Yuqing Hu et al.^14^ evaluated the interface shear strength between *UHPC* and *NSC*. Flexural strengthening using CFRP-Reinforced *UHPC* overlays was investigated in the study presented by Kadhim et al.^15^. Furthermore, hybrid *UHPC-NSC* beams Systems were addressed by Kadhim et al.^16^ and Wissam Nadir et al.^17^.

Focusing on *UHPC*, Yuqing Hu et al.^18^ assessed the shear strength of studs embedded in *UHPC*. In addition, Jia-Xing Huang et al.^19^ predicted the bond strength between fibers and the matrix in *UHPC*. Yuqing Hu et al.^20^ further presented an experimental and theoretical study on cracking strength of *UHPC* anchorage general zones. Furthermore, the torsional behavior of *UHPC* beams has also been studied by Zhou et al.^21^, Mitobaba et al.^22^, Kwahk et al.^23^, Ibrahim et al.^24^ and Fehling et al.^25^.

With respect to openings, Jabbar et al.^26^ studied the effect of openings on *HSC* and *UHPC* beams under flexural, torsional and cyclic loading. Later, Ye et al.^27^ and EL-Basiouny et al.^28^ discussed the combined effect of prestressing and openings on the behavior of reinforced normal strength and *HSC* beams, respectively. Moreover, Elsayed et al.^7^ investigated the shear behavior of *UHPC* beams with openings. Recently, the torsional strength of *UHPC* beams with openings has been investigated by Lina et al.^29^.

Despite the growing body of research on *UHPC* and openings, none of the aforementioned studies has investigated the influence of openings on the torsional behavior of pre-compressed *UHPC* hollow beams.

## Research significance

Hollow *UHPC* sections subjected to compression pre-force and torsion reflects realistic conditions in modern structural applications, such as bridge box girders, prestressed members and high-rise tubular columns, where axial forces precede torsional effects. The presence of web openings further underscores the practical necessity of considering such conditions in design. The combined effect of compression pre-force, torsion and openings in *UHPC* members remains underexplored in literature, despite its practical relevance. Accordingly, the current study provides novel insights into the performance and design of *UHPC* beams under these complex and realistic conditions.

## Experimental program

This section aims at discussing, in depth, the conducted experimental work. This includes design of *UHPC* mix, formwork and steel cages preparation, mixing and casting of *UHPC*, compression and tension testing, input data for test specimens (geometry, longitudinal and transverse steel and opening dimensions) and test procedure (installation of beams, loading types and conditions and output results).

### *UHPC* mix design

The study presented by Yousef et al.^30^ provided valuable insights and guidance for developing the *UHPC* mix design used in the present work. The mix consists of Portland cement grade 52.5N, silica fume, crushed quartz powder, quartz sand filtered from impurities, superplasticizers, clean water and end-hooked steel fibers. Table 1 presents the mix proportion of *UHPC* for 1 m^3^ in kg units. To prevent congestion of fibers in *UHPC* mix and not to decrease its flowability, fiber volume fraction, *V*_*f*_, should be 2.0% or less, as recommended by Zhou et al.^21^. and Cao et al.^31^. In contrast to using steel fibers with straight ends, end-hooked steel fibers are used to increase the toughness and torsional strength of *UHPC* beams, Zhou et al.^21^. Table 2 illustrates main characteristics of the used steel fibers.

**Table 1:** Mix proportion for 1 m^3^ in kg units.

Cement	Silica fume	Quartz sand	Quartz powder	Superplasticizers	Water	Steel fibers
900	225	850	270	36	180	157

**Table 2:** Characteristics of used steel fibers.

Type	Volume fraction	$\frac{{{\text{Length}}}}{{{\text{diameter}}}}$	Elastic modulus, MPa	Tensile strength, MPa
End-hooked steel fibers	2%	43.75	200,000	1100

### Specimens preparation

**Fig. 1:**
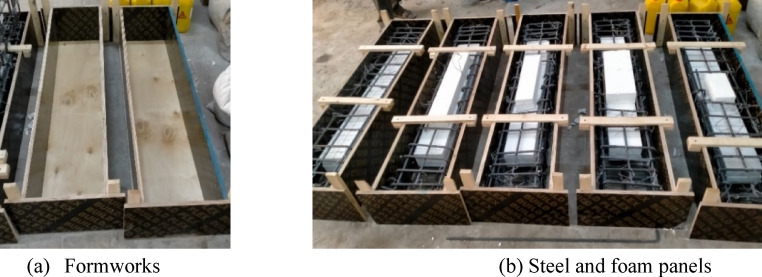
Specimen preparation.

The longitudinal and transverse steel were first prepared outside, and then placed in the equipped wooden formworks. During loading process, to measure strain in the longitudinal steel, one electrical strain gauge was installed at the mid-point of length of one of the lower longitudinal steel bars for each beam. Foam panels were used to create openings and hollow regions, Fig. 1.

### *UHPC* mixing and casting

**Fig. 2:**
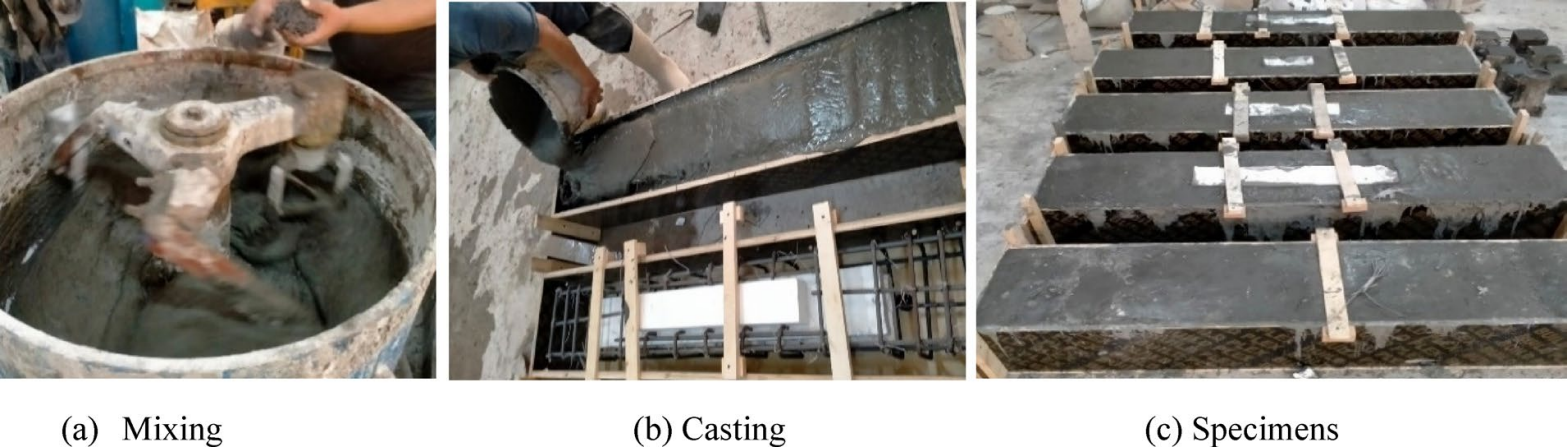
Mixing and casting of *UHPC* specimens.

At first, cement, silica fume, quartz sand and crushed quartz powder were added and dried mixed till the mix became homogenous. Thereafter, about three-quarters of plasticizers on water were progressively added to achieve an adequate fluidity and viscosity. This was followed by a gradual dispersing of the steel fibers into the mix and adding the remaining superplasticizers on water to give the suitable fluidity and workability of the mix. The total time of mixing was ranging from 10 to 12 min. Eventually, the mix was cast into the formworks. Furthermore, it was cast into standard cubes (100 × 100 × 100 mm) and cylinders of 100 mm dia. and 200 mm height to, later on, specify the mix characteristics. In the next day, the formworks and molds were demolded and the beams, cubes and cylinders were moisture cured for seven days. Figure 2 illustrates mixing and casting of *UHPC*.

### *UHPC* mechanical properties

**Fig. 3:**
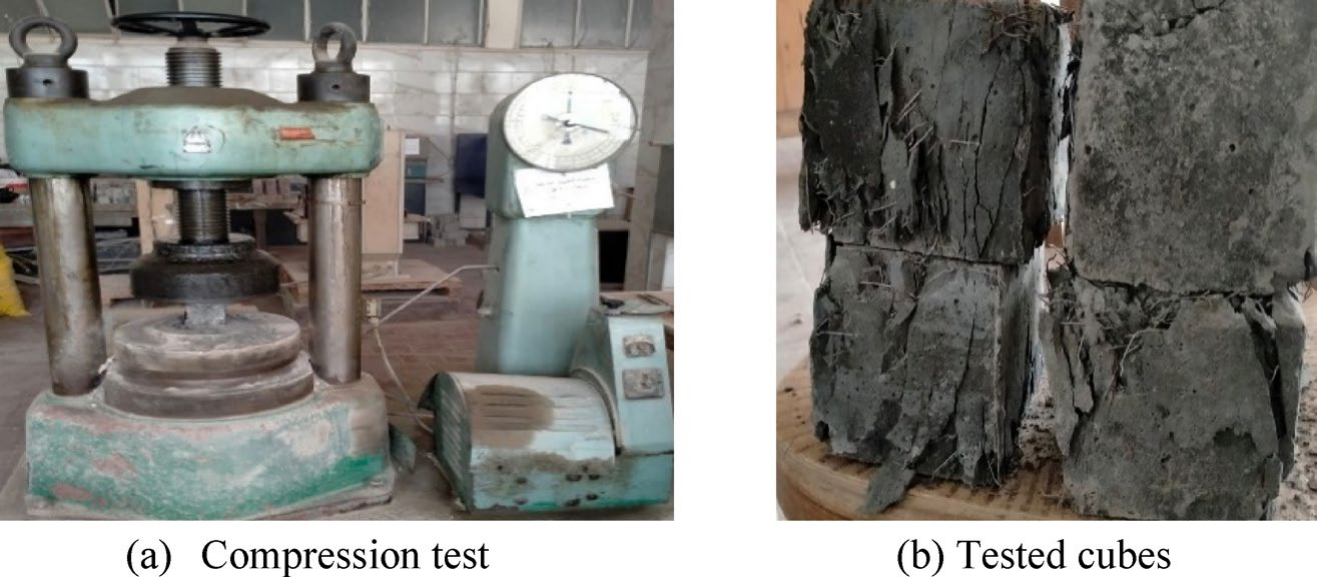
Compression test.

After seven days, the concrete compressive strength test was conducted for cubes and the average strength was 75 MPa. After ninety days, just before the testing of beams, the remaining cubes were tested in compression and the average strength $f_{cu }$ was 120 MPa. At the same time, the splitting test was conducted on the casted standard cylinders and the tensile strength $f_{t}$ was 13.50 MPa. Figure 3 illustrates testing of concrete cubes.

### General characteristics of specimens

**Fig. 4:**
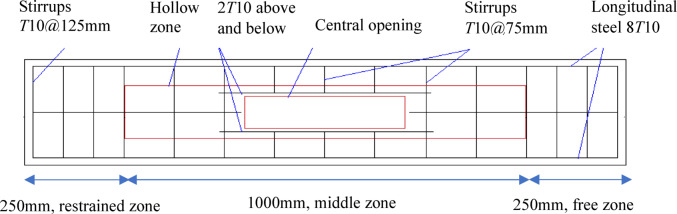
Geometry and steel arrangement of tested beams.

**Fig. 5:**
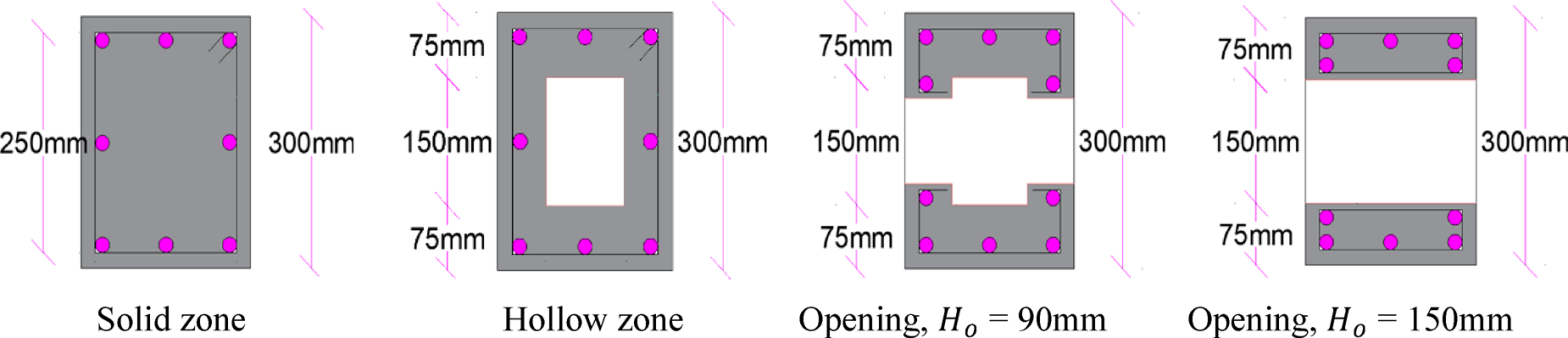
Cross sections.

Five *UHPC* pre-compressed hollow beams with and without openings were tested in the experimental program. Four beams with openings and one reference beam without openings. All beams had width, height *H* and length of 240, 300 and 1500 mm, respectively. The middle part of the span *L* (1000 mm) was hollowed, while right and left ends were solid (250 mm). One solid end was restrained, and thus preventing its rotation during the test, while the external torque was applied at the other solid end. In these two ends, intensified stirrups of 10 mm dia. and spaced closely at 70 mm were installed. Otherwise, in the middle zone, 10 mm dia. stirrups spaced at 125 mm were used. In all beams, the longitudinal steel was eight bars 10 mm dia. In four beams, a central opening has been created with length and height *L*_*o*_ and *H*_*o*_, respectively. The ratio $\frac{{L_{o} }}{L}$ was taken 0.20, 0.40 and 0.60, while $\frac{{H_{o} }}{H}$ was 0.30 and 0.50. Two longitudinal steel bars have been placed above and below each opening. Figure 4 presents the geometry and steel arrangement of tested *UHPC* beams, while Fig. 5 shows cross sections details. Tables 3 and 4 illustrate main characteristics of tested *UHPC* beams and reinforcing steel bars, respectively, while Table 5 presents steel reinforcement details.

**Table 3:** Characteristics of tested *UHPC* beams.

Beam	Width, mm	Height, mm	Length, mm	Section type	Opening *L*_*o*_ × *H*_*o*_, mm	$\frac{{L_{o} }}{L}$	$\frac{{H_{o} }}{H}$	$f_{cu}$, MPa	$f_{t}$, MPa
					Without opening				
B1	240	300	1500	Hollow, t = 75mm	200 × 90	0.20	0.30	120	13.50
B2									
B3					400 × 90	0.40	0.30		
B4					600 × 90	0.60	0.30		
B5					400 × 150	0.40	0.50		

where *t* is the web thickness of hollow beams.

**Table 4:** Characteristics and dimensions of reinforcing steel bars.

Diameter, mm	Cross-sectional area, mm^2^	Yield strength, MPa	Elastic modulus, MPa
10	78.5	350	200,000

**Table 5:** Steel reinforcement details.

Beam	Longitudinal steel		Steel around openings		Transverse steel		Reinforcement ratio^12^	
							Longitudinal, $\rho_{s}$%	Transverse, $\rho_{t}$%
	No	Dia., mm	Above	Below	End zones	Middle zone		
B1	8	10	–	–	*T*10@70mm	*T*10@125mm	1.05	1.17
B2			2*T*10	2*T*10				
B3								
B4								
B5								

where *T* is rebar diameter.

### Test setup

**Fig. 6:**
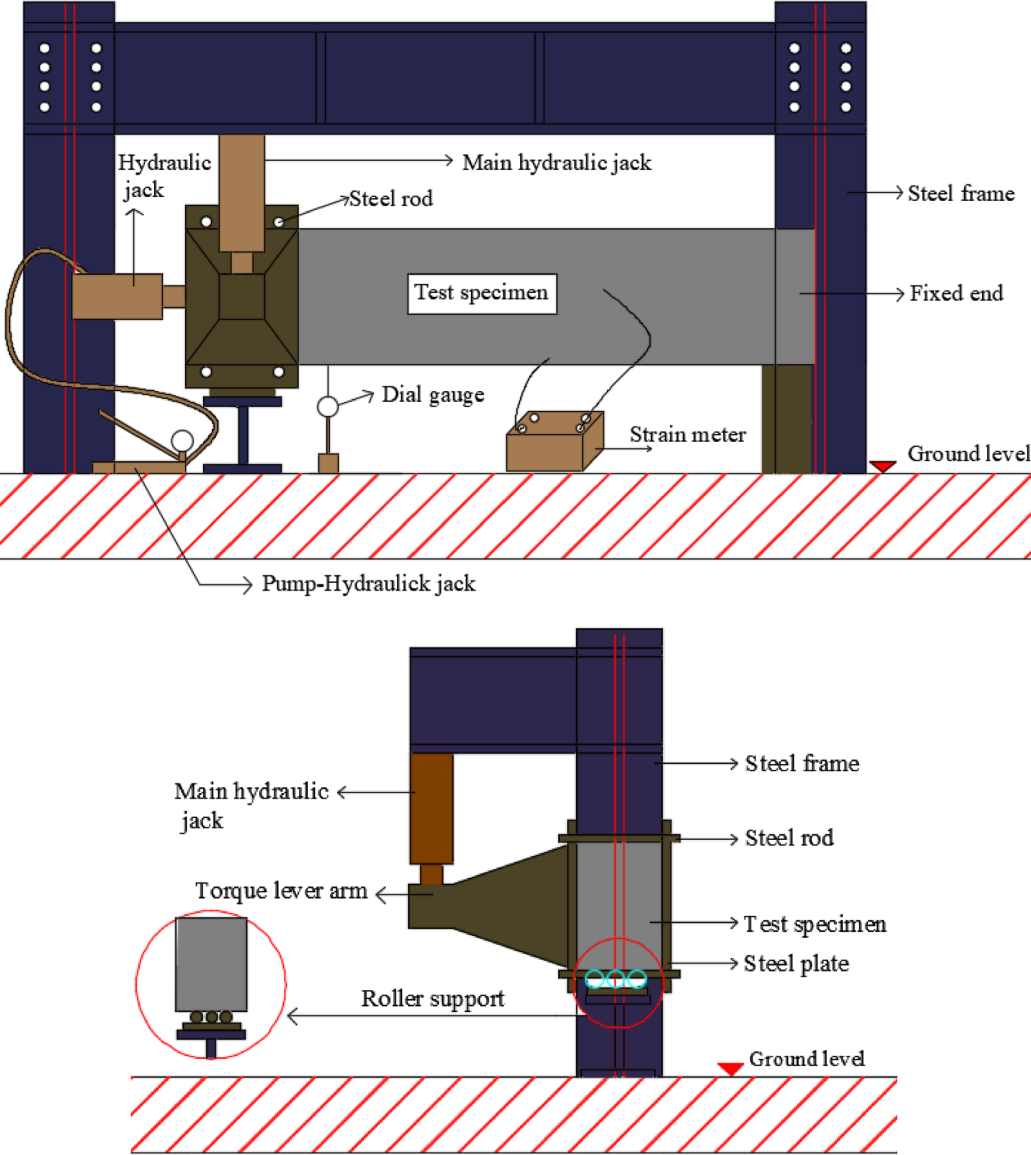
Schematic diagrams for test setup.

**Fig. 7:**
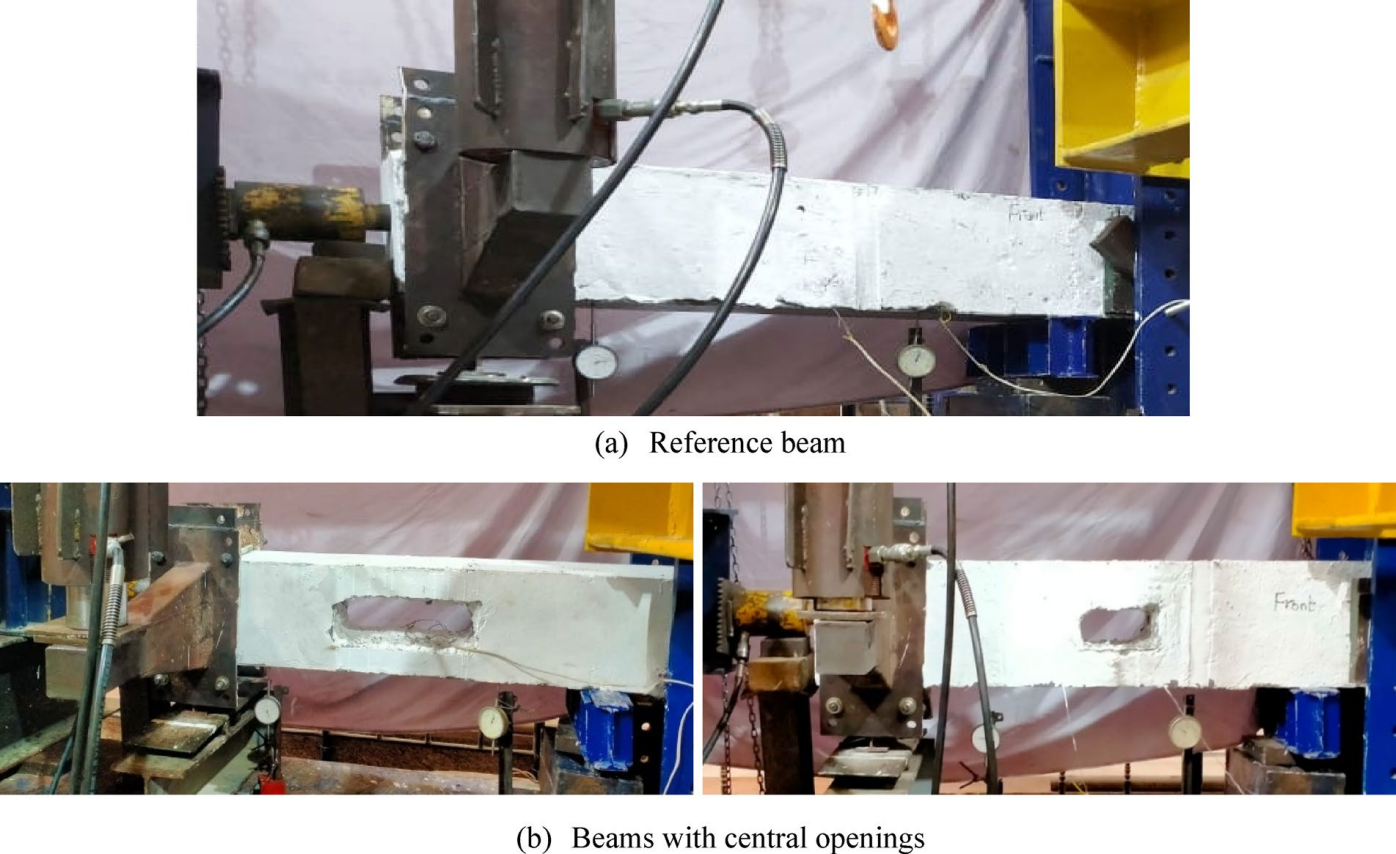
Test setup.

Figures 6 and 7 show the proposed loading system for conducting torsion test set-up, where one solid end was clamped using a vertical rigid steel frame to prevent rotation and the other solid end rested on rolling steel rods to freely rotate about its cross-section centroid. A rigid steel arm was installed at the edge of the front surface of each specimen. Another steel rectangular plate was installed at the edge of the rear surface of the specimen, corresponding to the rigid arm. The plate and the arm were then tied together using high strength steel rods, passing above and below the rotating end of each specimen, in order to rotate together as a one unit.

**Fig. 8:**
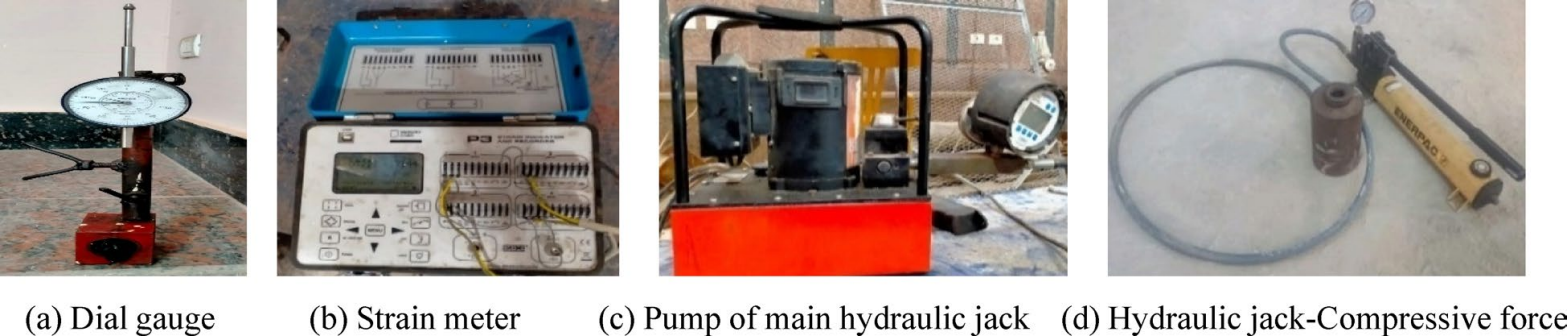
Used instruments.

To apply a compression pre-force to each specimen, a hydraulic jack (connected to a pump) was installed horizontally and centrally at the free side of each specimen. A strain meter was connected to strain gauges to gradually measure the strain of lower reinforcing steel bras during the test. A dial gauge was installed (tip of lower surface of each specimen, end of the middle zone) to gradually measure vertical displacement of each specimen at the specified point, and consequently determining the angle of twist $\theta$. $\theta$ was calculated using the tangent function, where tan $\theta = {\text{vertical displacement }}/{\text{beam width}}$. Here, the vertical displacement represents the opposite side, and the beam width represents the adjacent side of the right triangle formed by the beam deformation. Cracking was identified visually by monitoring the first surface cracks on white-painted beams during loading, and the corresponding torque was recorded. Figure 8 shows the used instruments. The twisting moment was applied by generating a vertical force using a giant hydraulic jack, connected to a pump, located at the end of rigid arm. The load increment was set at 20kN before *UHPC* cracking and reduced to 10kN after cracking. The lever arm of torque was varied from 450 to 500 mm.

## Experimental results and discussion

At first, a 150kN compression pre-force was applied to the tested *UHPC* beam using a hydraulic jack installed horizontally at its free side. After that, to exert on each tested beam by a torque, a multi-stage incremental load was applied at the end of the torque lever arm. At each load increment, all observations were monitored and recorded for each tested *UHPC* beam; cracking torque $T_{cr}$ , ultimate torque $T_{u}$ , cracking angle of twist $\theta_{cr}$, ultimate angle of twist $\theta_{u}$, cracking patterns and failure modes. These measurements helped among others, such as elastic stiffness $K_{1}$ , cracked torsional stiffness $K_{cr}$, post-cracking load-carrying capacity ${\varvec{\zeta}}_{{\varvec{t}}}$, ductility ${\varvec{\zeta}}_{{\varvec{\theta}}}$ and strain in lower reinforcing steel bars to investigate the effect of central openings (length and height) on the torsional behavior of *UHPC* beams.

### Ultimate torque

**Fig. 9:**
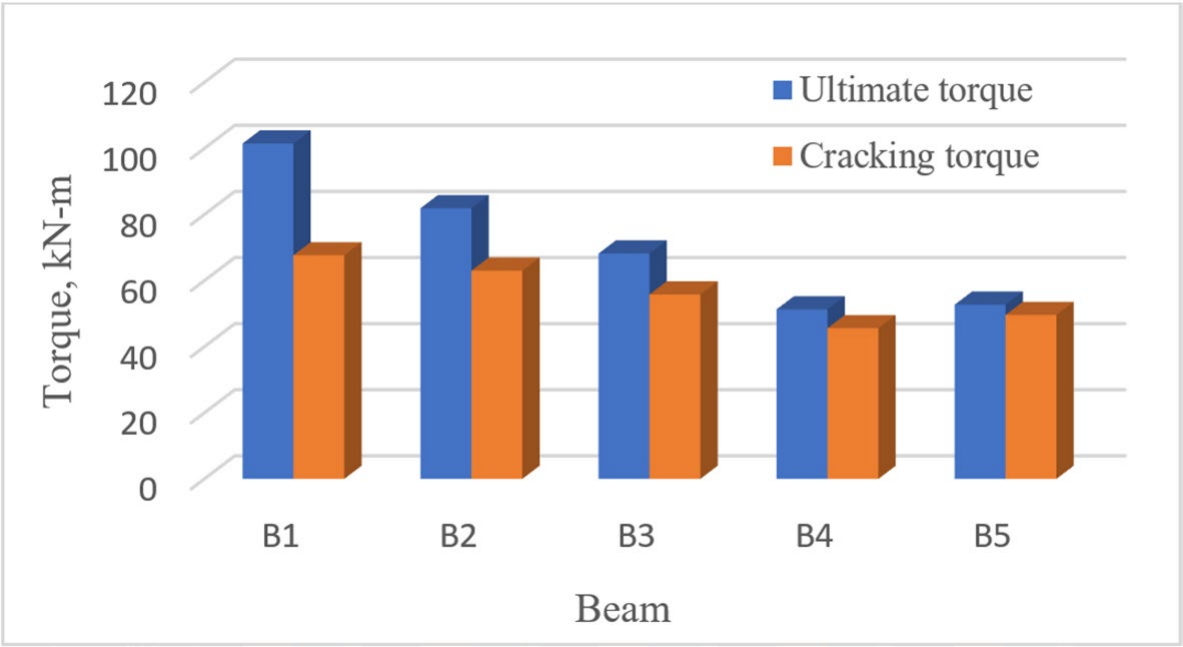
Ultimate- and cracking-torque.

With reference to Fig. 9 and Table 6, for the reference *UHPC* solid beam *B*1, the ultimate torque was 101.47 kN-m. It decreased to 81.90 k N-m, 68.20kN-m, 51.30kN-m and 52.70kN-m for beams *B*2, *B*3, *B*4 and *B*5, by 20%, 33%, 50% and 49%, respectively. This trend is expected, as an inverse correlation can be observed between the ultimate torque of the tested *UHPC* beams and the size of the web openings. Increasing the ratio $\frac{{L_{o} }}{L}$ to 0.40 for Beam *B*3 and 0.60 for Beam *B*4, compared to that of Beam *B*2 (0.20), decreased its ultimate torque by 17% and 38%, respectively. On the other hand, increasing the ratio $\frac{{H_{o} }}{H}$ from 0.30 for Beam *B*3 to 0.50 for Beam *B*5 led to a decrease in the ultimate torque of Beam *B*5 by 23%. These observations are confirmed by the numerically predicted stress distribution of the tested *UHPC* beams at failure (“Results comparison” section) and Fig. 21, which demonstrates that the reduction in ultimate torque is associated with increased stress values in the beams.

**Table 6:** Part of experimental results.

Beam	Cracking torque, $T_{cr}$, kN-m	Ultimate torque, $T_{u}$, kN-m	$\theta_{cr}$, rad-m^-1^	$\theta_{u}$, rad-m^-1^	$\zeta_{\theta }$	$\zeta_{t}$	$K_{1}$, kN-m^2^/rad	$K_{cr}$, kN-m^2^/rad
B1	67.65	101.475	0.0137	0.045	3.28	1.5	4937.95	1080.67
B2	63	81.90	0.0437	0.061	1.39	1.3	1441.65	1092.48
B3	55.80	68.20	0.051	0.0787	1.54	1.2	1094.12	447.65
B4	45.60	51.30	0.041	0.0833	2.03	1.12	1112.19	134.75
B5	49.60	52.70	0.041	0.075	1.83	1.06	1209.76	91.17

### Cracking torque

The influence of central opening on cracking torque was not as clear as it was with the ultimate torque. With reference to Fig. 9 and Table 6, the cracking torque of the reference beam *B*1 (67.65kN-m) was decreased by 7%, 17.5%, 32.5% and 27% for beams *B*2, *B*3, *B*4 and *B*5, respectively. Increasing this ratio $\frac{{L_{o} }}{L}$ to 0.40 for Beam *B*3 and to 0.60 for Beam *B*4, compared to that of Beam *B*2 (0.20) decreased the cracking torque by 11.5% and 27%, respectively. Eventually, the increase of the ratio $\frac{{H_{o} }}{H}$ from 0.30 for Beam *B*3 to 0.50 for Beam *B*5, decreased the cracking torque by 11%.

### Cracking patterns

**Fig. 10:**
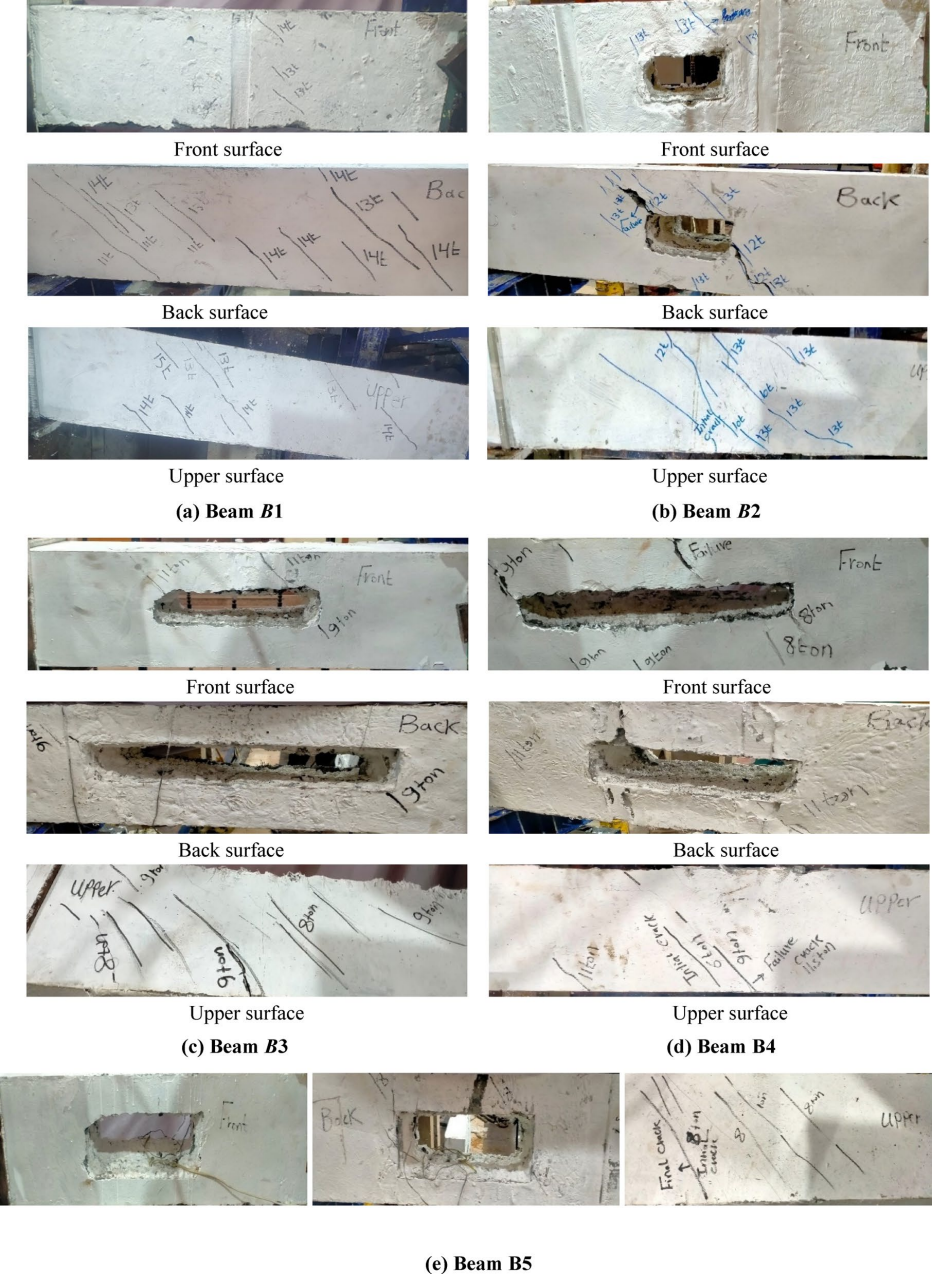
Modes of failure and cracking patterns.

The first crack was observed in the upper surface of each tested beam, excepting the reference beam, where the first crack was emerged in its back surface. The majority of cracks were observed in the reference beam, while there were few cracks in Beam *B*5, as its failure was rather a brittle one. In the middle zone, the inclination angle of cracks ranged from 40 to 52 degrees. Obviously, the torsional-shear failure was the predominant mode of failure for all beams. Figure 10 indicates cracking patterns in front, back and upper surfaces for all tested beams.

### Torsional stiffness

For *UHPC* beams, the torsional stiffness can be calculated in elastic and plastic stages. In the elastic stage, preceding the formation of cracks, the *UHPC* beams showed high elastic stiffnes $K_{1}$. In cotrast, in the plastic stage, the formation of cracks led to a decrease in the plastic stiffness $K_{cr}$. The torsional stiffness in elastic and plastic stages can be determined based on Cao et al.^31^ and Yang et al.^12^ euqations, repectively, as follows:

1 $$K_{1} = \frac{{T_{cr} }}{{\theta_{cr} }}$$

2 $$K_{cr} = \frac{{T_{u} - T_{cr} }}{{\theta_{u} - \theta_{cr} }}$$

**Fig. 11:**
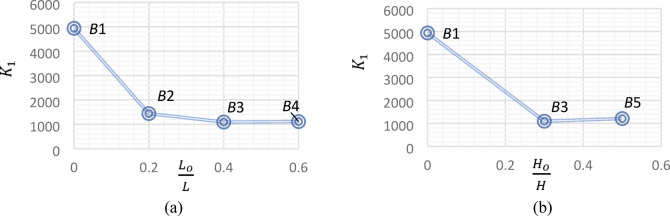
Effect of central opening dimensions on elastic stiffnes.

With reference to Fig. 11, the presence of the central opening in Beam *B*2 decreased its elastic stiffness by 70%, compared to the reference beam. However, increasing the ratio $\frac{{L_{o} }}{L}$ from 0.20 for Beam *B*2 to 0.40 and 0.60 for Beams *B*3 and *B*4, respectively, as in Fig. 11a, and the ratio $\frac{{H_{o} }}{H}$ from 0.30 for Beam *B*3 to 0.50 for Beam *B*5, as in Fig. 11b, had a minor effect on the elastic stiffness of the tested *UHPC* beams, where the elastic stiffness values of beams *B*3, *B*4 and *B*5 were rather equal.

**Fig. 12:**
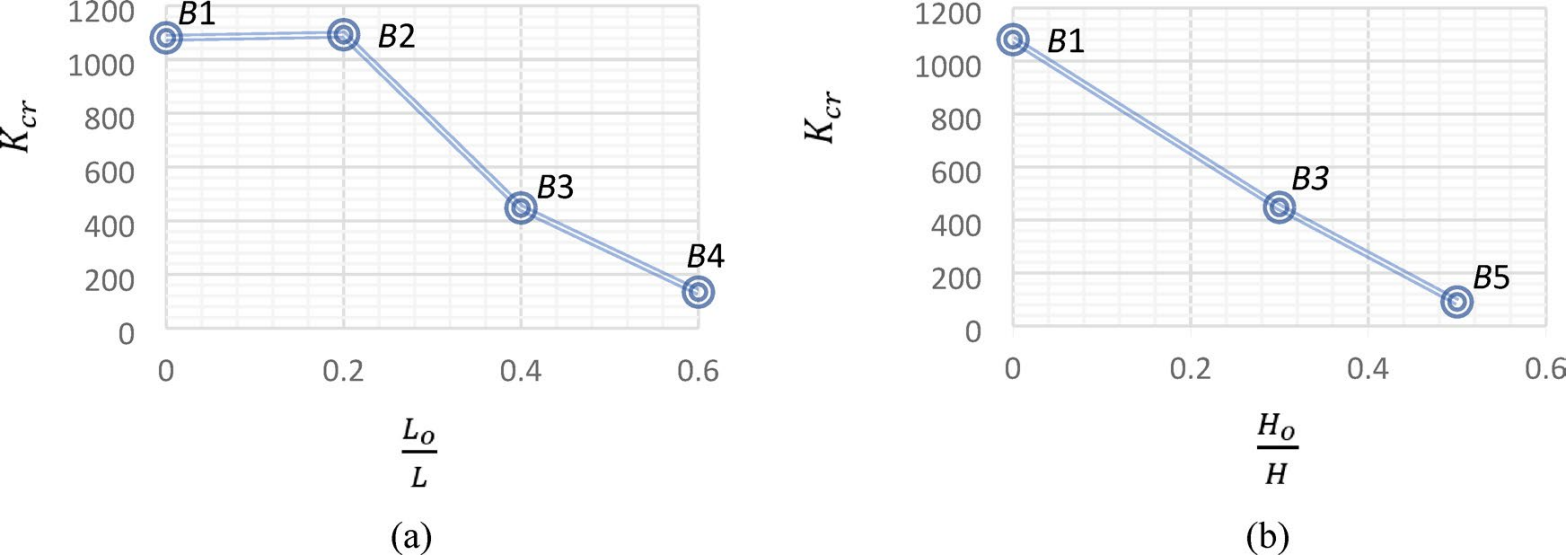
Effect of central opening dimensions on plastic stiffness.

In contrast to the elastic stiffness, the influence of central openings on the plastic stiffness was completely different. As Fig. 12 illustrates, creating a central opening in Beam *B*2 ($\frac{{L_{o} }}{L}$ = 0.20) had a negligible effect on its plastic stiffness, compared to the reference beam. On the other hand, increasing the ratio $\frac{{L_{o} }}{L}$ from 0.20 for Beam *B*2 to 0.40 for Beam *B*3 and to 0.60 for Beam *B*4, decreased the plastic stiffness by 59% and 88%, respectively. Also, the increase of the ratio $\frac{{H_{o} }}{H}$ from 0.30 for Beam *B*3 to 0.50 for Beam *B*5, led to a decrease in the plastic stiffness by 80%.

### Post-cracking load-carrying capacity

According to Zhou et al.^21^ assumptions, the post-cracking load-carrying capacity of *UHPC* beams, $\zeta_{t}$, can be calculated based on Eq. (3), as follows:

3 $$\zeta_{t} = \frac{{T_{u} }}{{T_{cr} }}$$

**Fig. 13:**
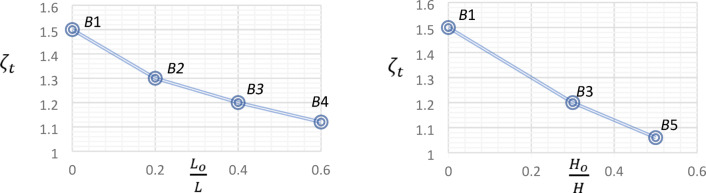
Effect of central opening dimensions on post-cracking load-carrying capacity.

The value of $\zeta_{t}$ for all tested *UHPC* beams is as presented in Table 6. Figure 13 illustrates the effect of central opening dimensions on the post-cracking load-carrying capacity.

As shown in Fig. 13 and Table 6, the presence of central opening in Beam *B*2 reduced its post-cracking load-carrying capacity by 14%, compared to the reference beam. Additionally, the increase in the ratio $\frac{{L_{o} }}{L}$ from 0.20 for Beam *B*2 to 0.40 and 0.60 for beams *B*3 and *B*4, reduced the post-cracking load-carrying capacity by 10% and 14%, respectively. Also, the increase in the ratio $\frac{{H_{o} }}{H}$ from 0.30 for Beam *B*3 to 0.50 for Beam *B*5, led to a reduction in its post-cracking load-carrying capacity by 12%.

### Torsional ductility

Torsional ductility of *UHPC* beams, $\zeta_{\theta }$, can be deduced based on Zhou et al.^21^ equation, as follows:

4 $$\zeta_{\theta } = \frac{{\theta_{u} }}{{\theta_{cr} }}$$

**Fig. 14:**
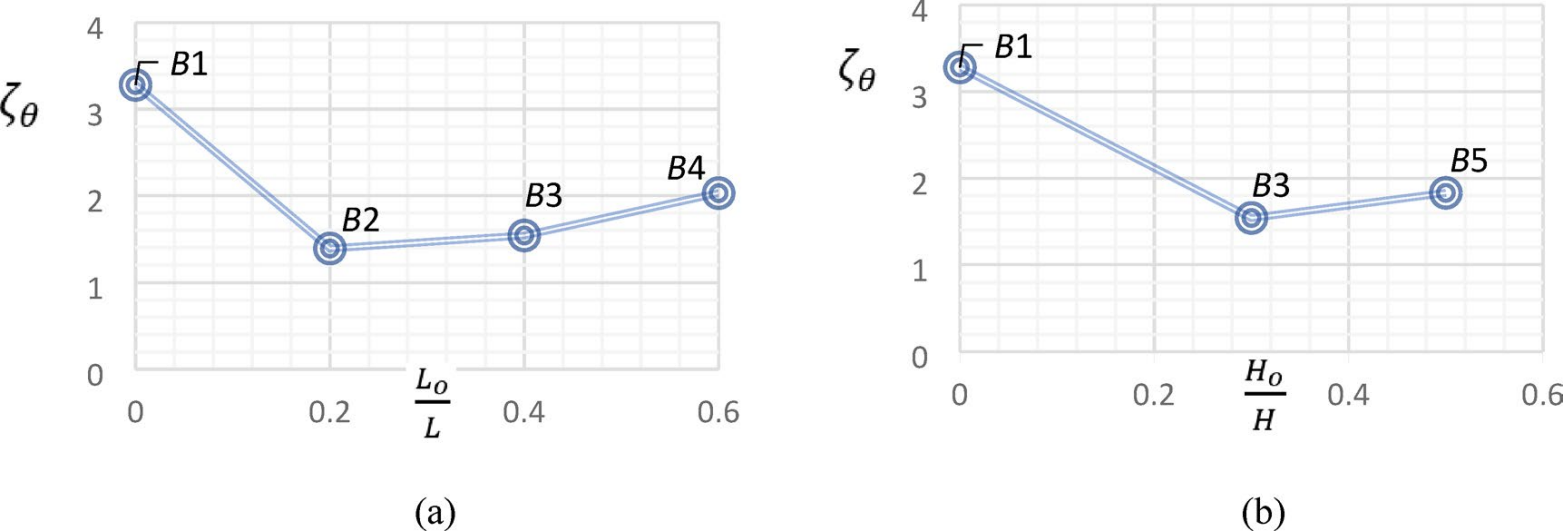
Effect of central opening dimensions on torsional ductility.

Table 6 and Fig. 14 illustrate the resulted values of $\zeta_{\theta }$ for all the tested *UHPC* beams.

With reference to Table 6 and Fig. 14, the torsional ductility of Beam *B*2 was 42% compared to that of Beam *B*1. This was due to the presence of the central opening. Increasing the central opening length of Beam *B*2 ($\frac{{L_{o} }}{L}$ = 0.20), led to an unexpected increase in its torsional ductility by 11% and 46%, in comparison with beams *B*3 ($\frac{{L_{o} }}{L}$ = 0.40) and *B*4 ($\frac{{L_{o} }}{L}$ = 0.60), respectively. A similar behavior was noticed in the beams having the same central opening length but different height. The torsional ductility of *B*eam *B*3 ($\frac{{H_{o} }}{H}$ = 0.30) was 84% of that of Beam *B*5 ($\frac{{H_{o} }}{H}$ = 0.50).

### Strain in lower reinforcing steel bars

**Fig. 15:**
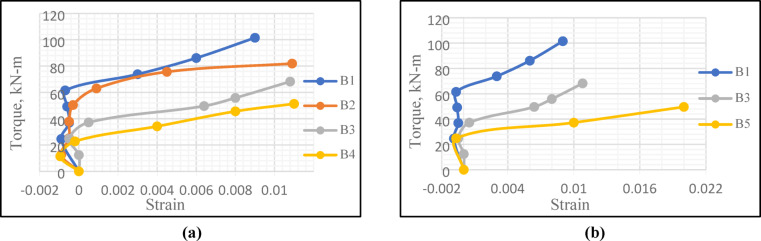
Effect of central opening dimensions on strain in lower bars.

As illustrated in Fig. 15, at the initial loading stages of all tested beams, the lower reinforcing steel bars showed negative strain values, due to the influence of compression pre-force on the *UHPC* beams. Furthermore, at the same load level, the reference beam showed less strain values than those observed in all other tested beams.

A direct proportionality was emerged between central opening length and strain values in lower reinforcing steel bars. At the same level of loading, Beam *B*4 ($\frac{{L_{o} }}{L}$ = 0.60) showed higher strain values than those in Beam *B*3 ($\frac{{L_{o} }}{L}$ = 0.40) which showed higher strain values than those in Beam *B*2 ($\frac{{L_{o} }}{L}$ = 0.20), Fig. 15a. The direct proportionality was also noticed between central opening height and values of strain, as Beam *B*5 ($\frac{{H_{o} }}{H}$ = 0.50) showed higher values of strain than those noticed in Beam *B*3 ($\frac{{H_{o} }}{H}$ = 0.40), Fig. 15b.

### Torque–angle of twist curve

**Fig. 16:**
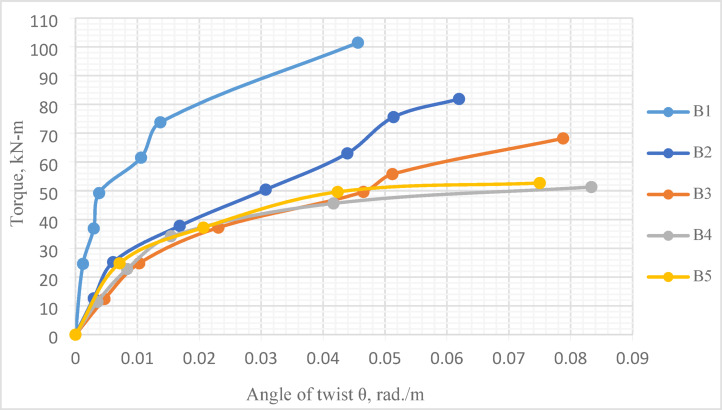
Torque–angle of twist curves for all tested beams.

The applied torque–angle of twist curve for the tested *UHPC* beams is shown in Fig. 16. Obviously, at all loading stages, the recorded angles of twist for Beam *B*1were less than those noticed in beams with central openings. Furthermore, at the same load level, increasing either height or length of central opening, increased the angle of twist for each tested beam.

Figure 16 and Table 6 illustrate the minor effect of increasing central opening height on ultimate angle of twist, $\theta_{u}$, where it was approximately the same for beams *B*3 and *B*5, which have different central opening heights. In contrast, the effect of increasing central opening length was apparent, as $\theta_{u}$ for Beam *B*2 ($\frac{{L_{o} }}{L}$ = 0.20) was 1.29 and 1.36 times that of Beam *B*3 ($\frac{{L_{o} }}{L}$ = 0.40) and Beam *B*4 ($\frac{{L_{o} }}{L}$ = 0.40), respectively. The same result is next numerically verified in “Ultimate torque and angle of twist” section.

## Numerical study

Recently, finite element (*FE*) modeling of concrete elements, an economic and precise method, has been become indispensable. The current numerical investigation was conducted with Abaqus software^32^. At first, validation of the numerical models has been achieved through the *FE* analysis of the experimentally tested *UHPC* five beams and comparing the obtained results with the experimental results. Then, the numerical analysis has been enlarged through modeling other *UHPC* beams with openings and presenting their torsional behavior in terms of torsional strength, torque–angle of twist relation and other output results that could not be experimentally obtained such as the curve between the applied torque and strain in concrete for the modeled *UHPC* beams.

### Finite element parameters

In comparison with conventional concrete, the presence of fine particles and fibers in *UHPC* and being free from aggregate changes its characteristics in tension, compression, shear and torsion^22^. Based on Fakeh et al.^33^, in Abaqus software, the concrete damage plasticity (*CDP*) model can be considered an appropriate model to represent *UHPC* material.

The stress–strain curve for *UHPC* concrete in compression consists of ascending and descending parts. Upon reviewing various existing models (Yan^34^, Zhao et al.^35^, Wang et al.^36^ and Graybeal^37^), it was concluded by Fakeh et al. ^33^ that the best simulating model for the stress–strain curve for *UHPC* is that presented by Graybeal ^37^ for the ascending part and by Prem et al.^38^ for the descending part. The *UHPC* elastic modulus $E_{c}$ was calculated utilizing the formula proposed by Grybeal^37^. The stress–strain curve for *UHPC* in tension was derived based on the equations developed by In-Hwan et al.^39^.

The prerequisites of the *CDP* model were considered as illustrated in Table 7, where the dilation angle and the stress ratio were considered 55 and 3, respectively. These values were considered based on Fakeh et al.^33^ recommendations, where multiple axial compression tests concluding the most accurate inputs for *UHPC* were presented.

**Table 7:** *CDP* parameters, Fakeh et al.^33^.

Dilation angle (Ψ)	55
Eccentricity	0.10
Stress ratio f_b0_/f_c0_	3
Viscosity parameter	0.001

Additionally, damage parameters ($d_{c} {\text{and}} d_{t}$) were considered to define the stiffness degradation in the descending parts for the compressive and tensile stress–strain curves respectively, based on Kadhim et al.^40^ recommendations as follow:

5 $$d_{c} {\text{or}} d_{t} = 1 - \frac{\sigma }{f}$$

where σ is the actual compressive or tensile stress and *f* is the *UHPC* compressive or tensile strengths.

The reinforcing steel was defined as (Deformable wire) with yield strength, density, modulus of elasticity and Poisson ratio of 350 MPa, 7800 kg/m^3^, 200000 MPa and 0.30, respectively. The stress–strain curve for steel in tension has been used based on Zhu et al.^41^ recommendations. In addition, to establish a proper interaction between *UHPC* and the reinforcing steel, the steel was embedded within the concrete matrix using the (Embedded region) technique in Abaqus software.

**Fig. 17:**
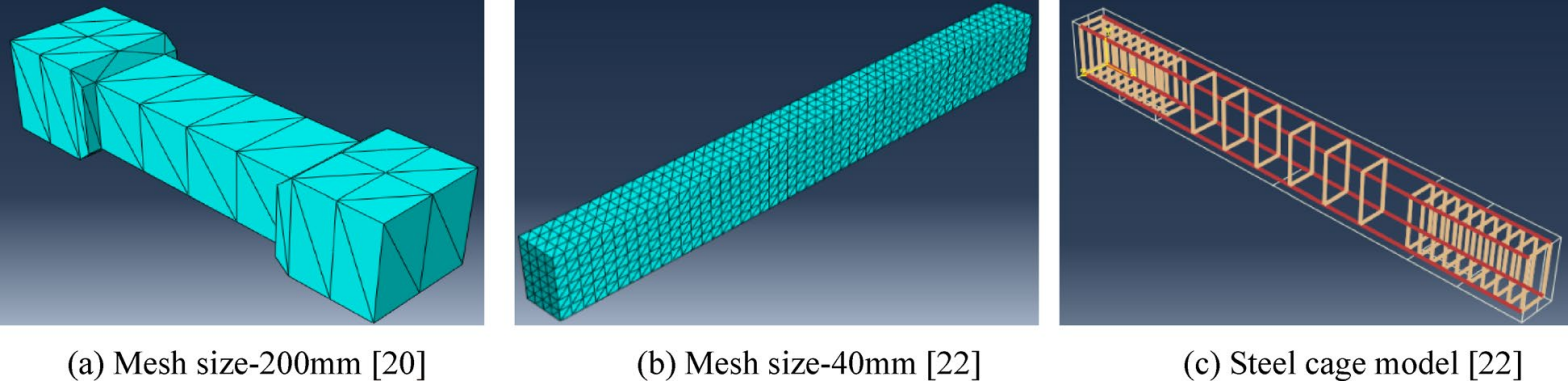
Finite element models for previously tested *UHPC* beams. Figure generated using Abaqus 6.14-1 (SIMULIA, Dassault Systèmes). Available at*:* https://www.3ds.com/products-services/simulia/products/abaqus.

To choose an appropriate mesh size for simulating the *UHPC* beams, two *UHPC* beams which tested in two previous experimental related studies, were modeled using the *FE* method with Abaqus software; Beam L121S084F150 by Cao et al.^31^ and Beam UPF1(0.9)28 by Fehling et al.^25^. Four different mesh sizes were checked for each beam; 200, 150, 60, and 40 mm. Figure 17 shows the *FE* models of the two beams and the output results are presented in Table 8.

**Table 8:** Experimental and *FE* torsional strength with different mesh sizes.

References	Torsional strength, kN-m				
	*FE*				Exp
	Mesh size, mm				
	40	60	150	200	
^31^	14.82	21.41	27.72	29.55	21.90
^25^	42.33	42.42	42.86	48.18	46.32

The results of the two beams, shown in Table 8, proved that the selected parameters of the *CDP* model produced better results with mesh size of 60 mm. Similar mesh size was recommended in El-Basiouny et al.^42^.

### Finite element-based models

Based on the aforementioned *FE* parameters, ten *UHPC* beams were modeled with Abaqus software. The beams included the five tested beams in this study (*B*1 to *B*5), in addition to other five beams (*B*6 to *B*10) with the same characteristics which previously presented in Tables 3, 4 and 5, but with different values for the ratios $\frac{{L_{o} }}{L}$ and $\frac{{H_{o} }}{H}$, as presented in Table 9.

**Table 9:** Input data of tested *UHPC* beams.

Beam	Opening ($L_{o}$ by $H_{o}$), mm	$\frac{{L_{o} }}{L}$	$\frac{{H_{o} }}{H}$	Beam	Opening ($L_{o}$ by $H_{o}$), mm	$\frac{{L_{o} }}{L}$	$\frac{{H_{o} }}{H}$
B1	Without opening	–	–	B6	200 × 150	0.20	0.50
B2	200 × 90	0.20	0.30	B7	600 × 150	0.60	0.50
B3	400 × 90	0.40	0.30	B8	200 × 210	0.20	0.70
B4	600 × 90	0.60	0.30	B9	400 × 210	0.40	0.70
B5	400 × 150	0.40	0.50	B10	600 × 210	0.60	0.70

To accurately simulate the experimental work, a roller-fixed support setup was adopted to simulate boundary conditions.

**Fig. 18:**
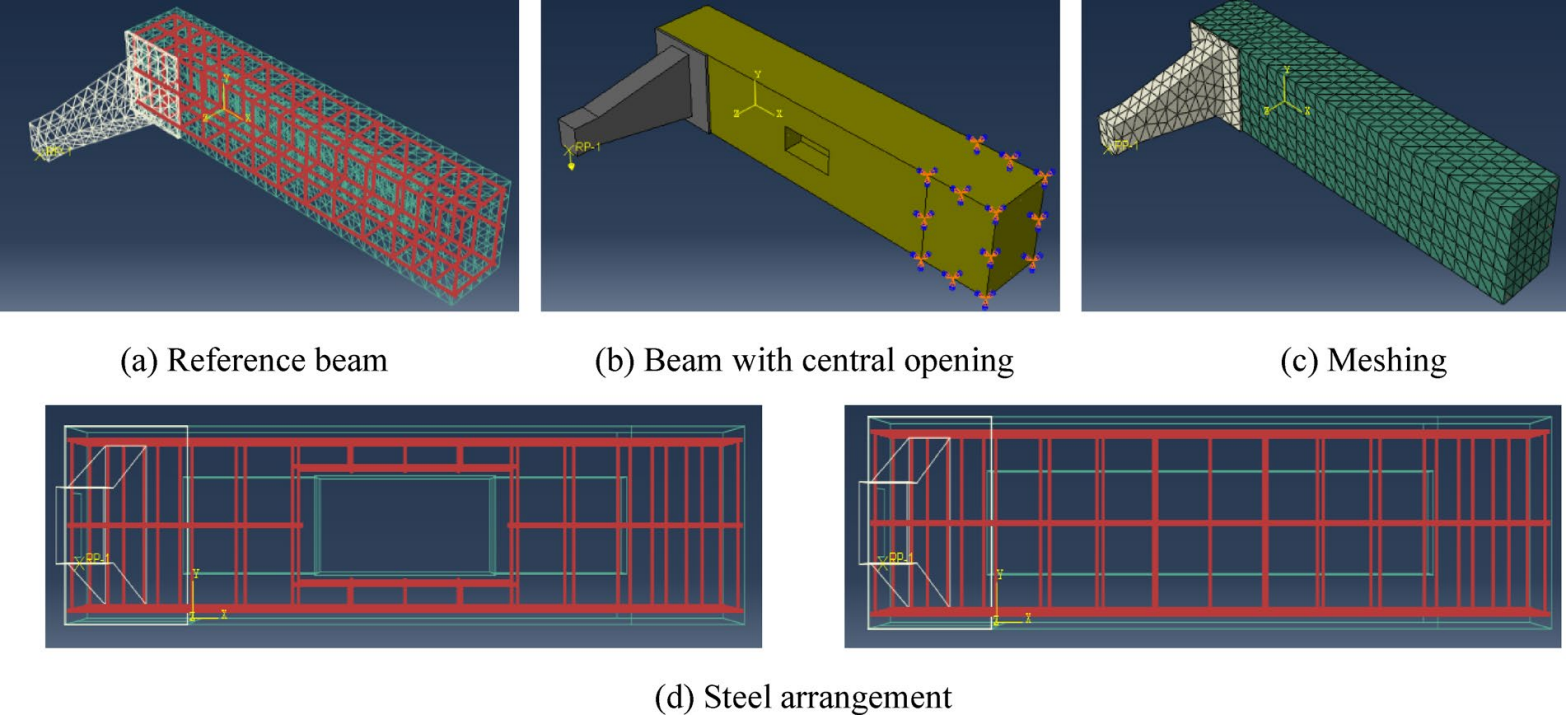
Schematic representation of finite element models. Figure generated using Abaqus 6.14-1 (SIMULIA, Dassault Systèmes). Available at*:* https://www.3ds.com/products-services/simulia/products/abaqus.

Due to the complex geometry of the lever arm, (Tet) mesh elements were employed, as (Hex) elements would require extensive partitioning and compromise mesh quality. (Tet) mesh convergence was previously verified, demonstrating high accuracy, as shown in Tables 8 and 10. Figure 18 illustrates a schematic representation of the *FE* models.

**Table 10:** Experimental and *FE* ultimate torque.

Beam	$T_{u}^{FE}$, kN-m	$T_{u}^{Exp.}$, kN-m	$\frac{{T_{u}^{FE} }}{{T_{u}^{Exp.} }}$
B1	100.56	101.475	0.99
B2	73.82	81.90	0.90
B3	61.21	68.20	0.90
B4	50.87	51.30	0.99
B5	51.56	52.70	0.97
Mean			0.95

## Output results

To evaluate the numerical and experimental output results, the numerical results of the *UHPC* beams (*B*1 to *B*5) are next displayed and compared to the experimental results. Thereafter, the torsional behavior of the *UHPC* ten beams (*B*1 to *B*10) is presented with introducing some output results that could not be experimentally obtained.

### Results comparison

**Fig. 19:**
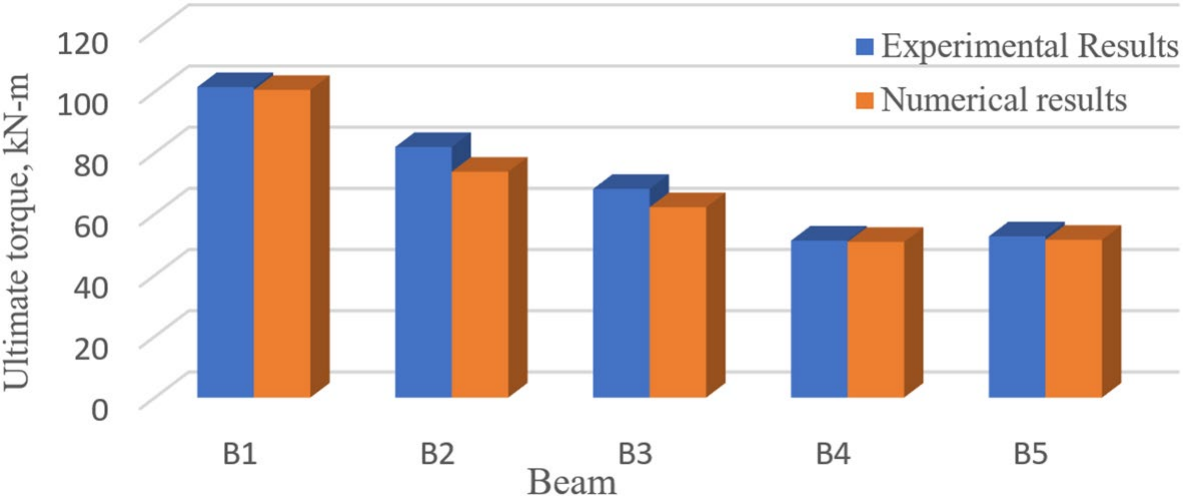
Experimental and *FE* ultimate torque, kN-m.

**Fig. 20:**
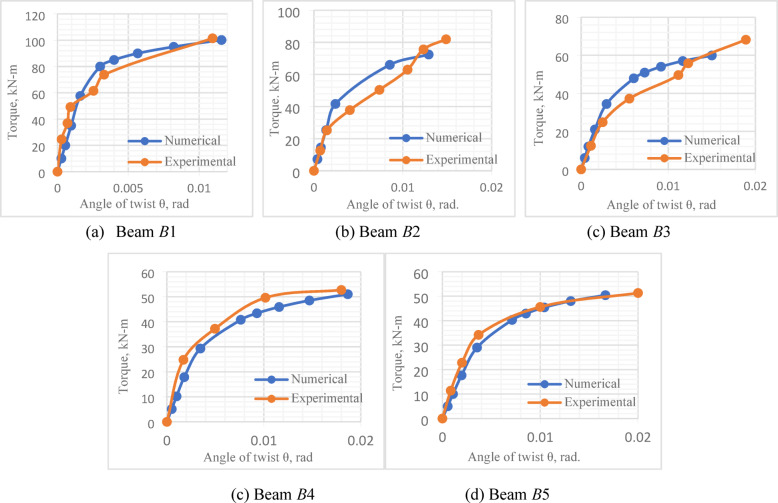
Experimental and numerical torque–angle of twist curve for Beams *B*1 to *B*5.

Figure 19 and Table 10, on one hand, present a comparison between the numerical and experimental ultimate torque for the tested *UHPC* five beams. Figure 20, on the other hand, gives a comparison between the numerical and experimental torque–angle of twist curve.

**Fig. 21:**
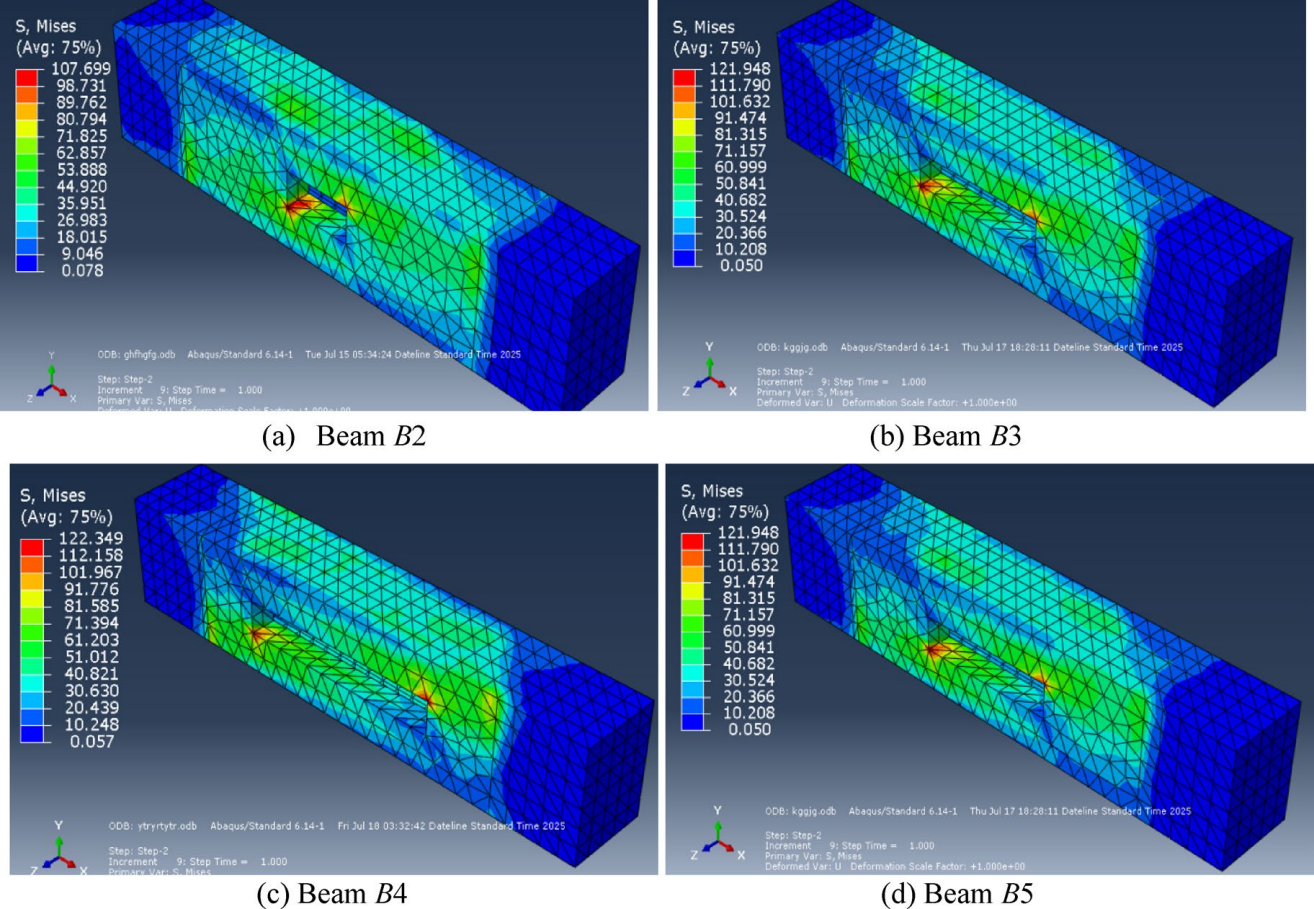
Stresses distribution in *UHPC* beams at failure. Figure generated using Abaqus 6.14-1 (SIMULIA, Dassault Systèmes). Available at*:* https://www.3ds.com/products-services/simulia/products/abaqus.

As illustrated in Table 10 and Fig. 19, the comparison between the experimental and numerical torsional strength, for the first *UHPC* five beams, was most satisfactory, with a mean value of 0.95. Furthermore, the experimental and numerical torque–angle of twist curves were in good agreement, Fig. 20. Figure 21 illustrates the stress distribution in the modeled beams, revealing high stress concentrations around the openings. These elevated stress levels are closely associated with the reduction in the ultimate torque capacity of the beams. In contrast, negligible stress values are observed near both beam ends, indicating minimal torsional demand in those regions.

### Numerical results

#### Ultimate torque and angle of twist

**Fig. 22:**
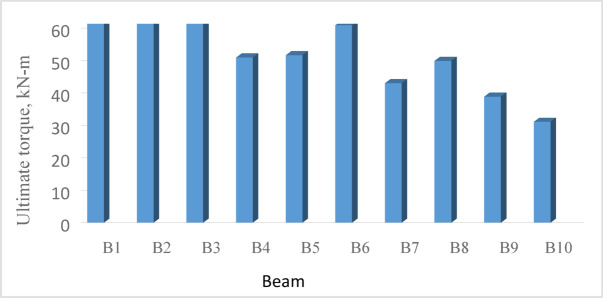
Numerical ultimate torque for beams *B*1 to *B*10.

**Fig. 23:**
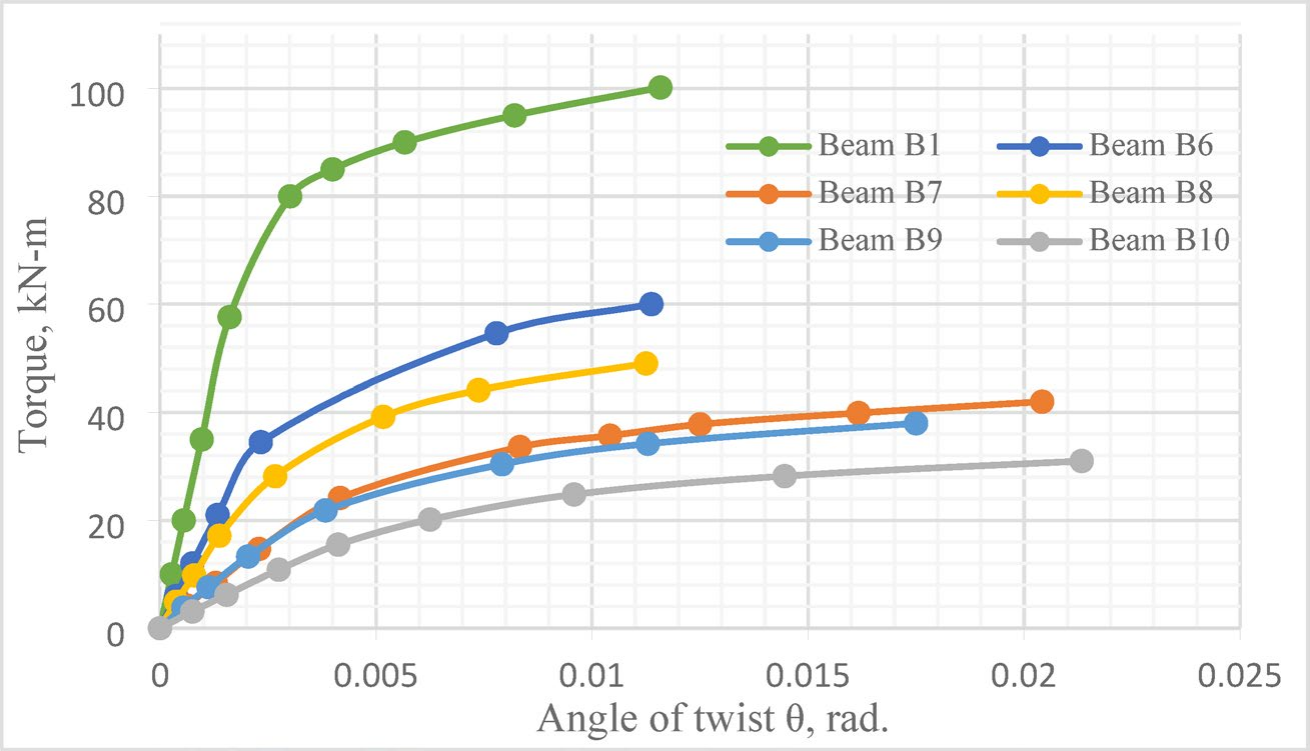
Torque–angle of twist curve for the reference beam *B*1 and beams *B*6 to *B*10.

The numerical results of the tested *UHPC* ten beams (*B*1 to *B*10) are presented in Figs. 22 and 23 and Table 11, in terms of ultimate torque, ultimate angle of twist and torque–angle of twist curve.

**Table 11:** Numerical ultimate torque for beams *B*1 to *B*10.

Beam	$T_{u}^{FE}$, kN-m	$\theta_{ult}^{FE}$
B1	100.56	0.0116
B2	73.82	0.0129
B3	61.21	0.015
B4	50.87	0.0167
B5	51.56	0.0186
B6	60.38	0.0114
B7	42.95	0.020
B8	49.85	0.01125
B9	38.80	0.0175
B10	31.04	0.021

As illustrated in Table 11, compared to the reference beam, introducing a central opening, for Beam *B*6, with $\frac{{{\varvec{L}}_{{\varvec{o}}} }}{{\varvec{L}}}$ = 0.20 and $\frac{{H_{o} }}{H}$ = 0.50, decreased the ultimate torque from 100.56 to 60.38 kN-m; about 40%. The reduction increased to 50%, 61% and %69, when the ratio $\frac{{{\varvec{L}}_{{\varvec{o}}} }}{L}$ was 0.20, 0.4 and 0.60 for Beams *B*8, *B*9 and *B*10, respectively.

The influence of central opening height on the ultimate angle of twist ${\varvec{\theta}}_{{{\varvec{ult}}}}^{{{\varvec{FE}}}}$ was not clearly apparent. For instance, beams *B*2, *B*6 and *B*8, having the same central opening length but different height, showed roughly similar values of ${\varvec{\theta}}_{{{\varvec{ult}}}}^{{{\varvec{FE}}}}$; of 0.0129, 0.0114 and 0.0112, respectively. The same behavior was noticed for beams *B*7 and *B*10, where ${\varvec{\theta}}_{{{\varvec{ult}}}}^{{{\varvec{FE}}}}$ was approximately the same, while it slightly decreased in Beam *B*4.

In contrast to the effect of central opening heigh the effect of its length on ${\varvec{\theta}}_{{{\varvec{ult}}}}^{{{\varvec{FE}}}}$ was clearly noticeable. In despite of the equal central opening height of beams (*B*2 and *B*4), (*B*6 and *B*7) and (*B*8 and *B*10), the value of ${\varvec{\theta}}_{{{\varvec{ult}}}}^{{{\varvec{FE}}}}$ increased by 30%, 75% and 86% for beams *B*4, *B*7 and *B*10, compared to beams *B*2, *B*6 and *B*8, respectively, due to the change in the ratio $\frac{{{\varvec{L}}_{{\varvec{o}}} }}{L}.$

#### Torque–angle of twist curve

The presence of central opening has an immense effect on the slope of the torque–angle of twist curve that was sharply decreased, particularly in beams *B*7, *B*9 and *B*10 compared with Beam *B*1, Fig. 23.

#### Strain in concrete

**Fig. 24:**
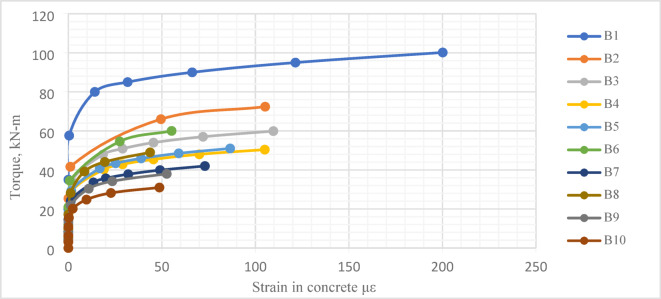
Torque-concrete strain curve.

The *FE* modeling was a feasible opportunity to enrich the study with some output results that could not experimentally measured, such as strain in concrete, Fig. 24. The average value of concrete strain in the upper surface of each tested beam was determined with Abaqus software.

**Fig. 25:**
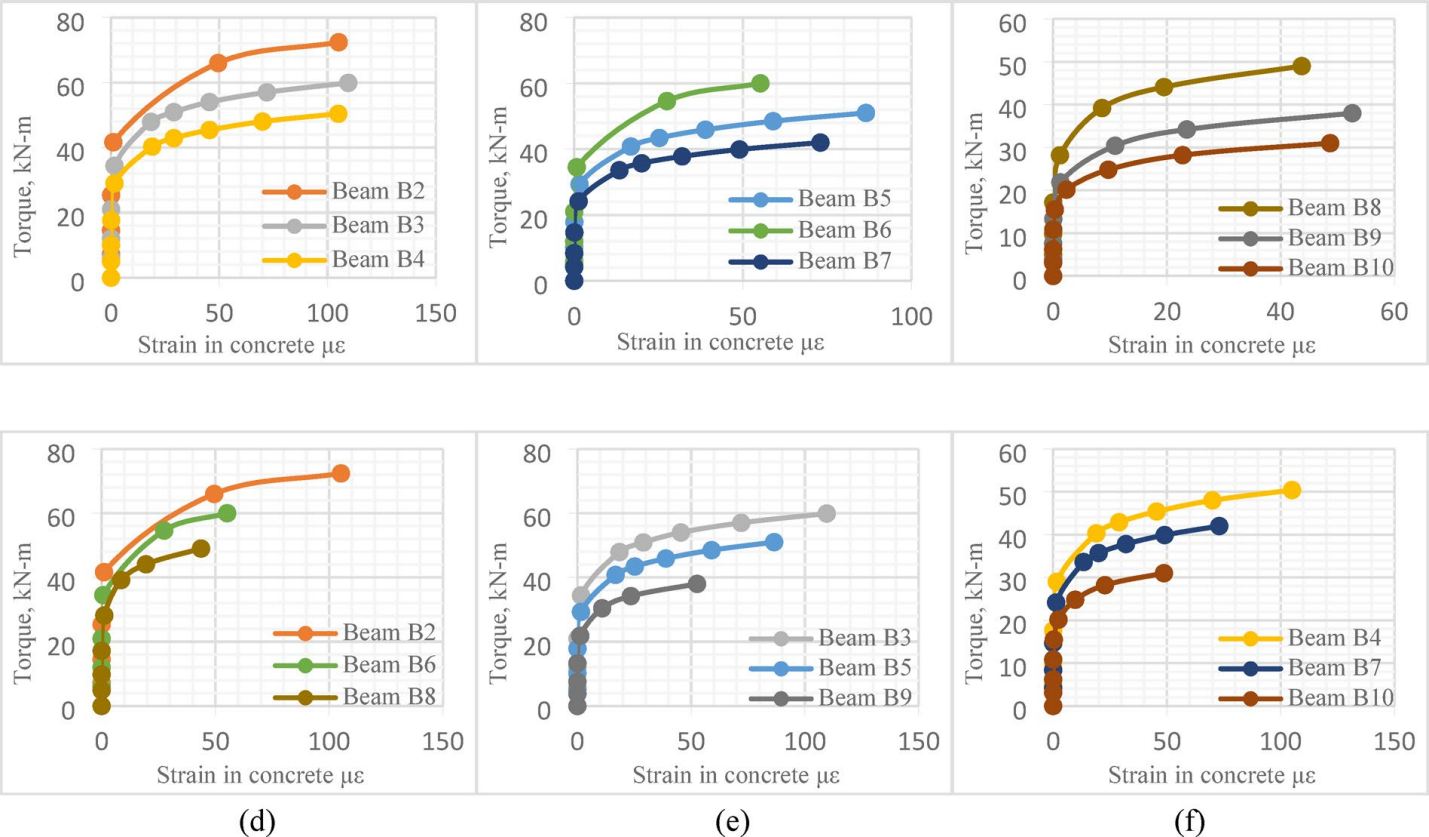
Effect of central opening dimensions on torque-concrete strain relation.

As shown in Fig. 25, the effect of central opening height, compared to its length, can be considered major. For instance, beams *B*2, *B*3 and *B*4, with the same central opening height and not same its length, illustrated almost equal values of the maximum concrete strain, although the ultimate torque was not, Fig. 25a. Similarly, for beams *B*8, *B*9 and *B*10, the maximum concrete strain was almost equal, Fig. 25c.

On the other side, increasing central opening height led to a noticeable increase in the maximum concrete strain. This was observed in beams having the same central opening length and not same its height, (*B*2 with *B*6 and *B*8), Fig. 25d, or beams (*B*3 with *B*5 and *B*9), Fig. 25e or beams (*B*4 with *B*7 and *B*10), Fig. 25f. The maximum concrete strain of the reference beam *B*1 was almost twice the ultimate strain of Beam *B*2.

#### Additional numerical models

**Fig. 26:**
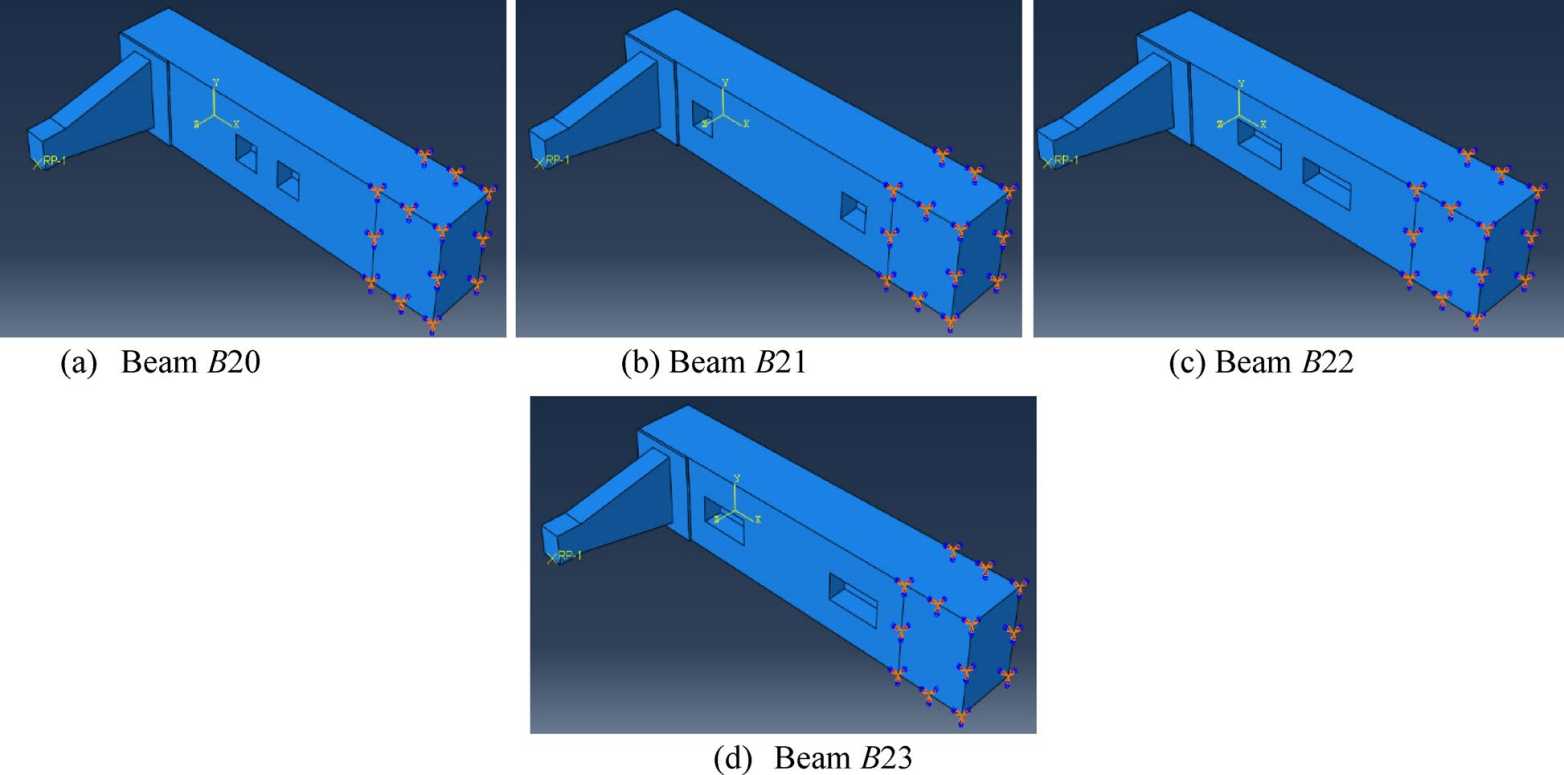
Schematic representation of finite element models. Figure generated using Abaqus 6.14-1 (SIMULIA, Dassault Systèmes). Available at: https://www.3ds.com/products-services/simulia/products/abaqus.

**Fig. 27:**
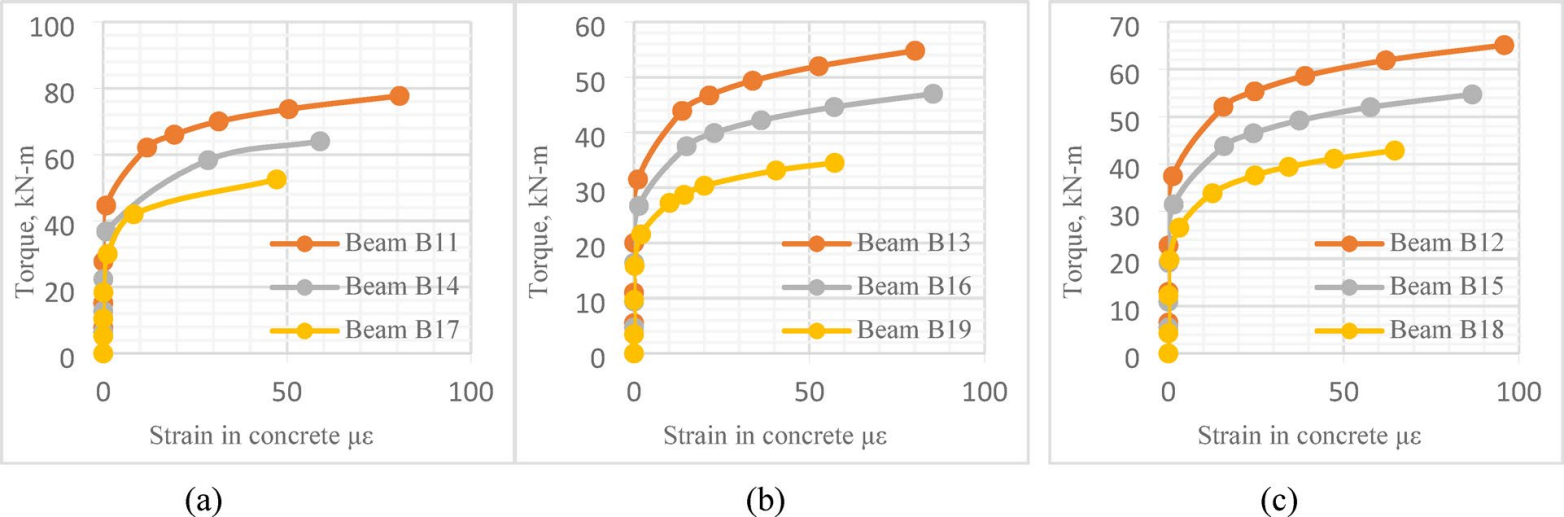
Effect of central opening length on torque-concrete strain relation.

For greater comprehensiveness and applicability, additional 13 *UHPC* beams with different key parameters were modeled using Abaqus software and incorporated into the study. Nine *UHPC* models (*B*11 to *B*19) were designed to replicate beams *B*2 to *B*10, respectively, in terms of geometry and material properties. However, they were subjected to a higher axial pre-compression load of 300kN instead of150kN.The other four additional *UHPC* beams (*B*20 to *B*23) were designed with two web openings instead of a single central one, with variations in both the position and dimensions of the openings. Beams *B*20 and *B*21 had openings measuring 100 × 90 mm^2^, whereas *B*22 and *B*23 featured larger openings of 200 × 90 mm^2^. In beams *B*21 and *B*23, the openings were positioned 100 mm from the beam ends, while in *B*20 and *B*22, the openings were located 50 mm from the beam center, as illustrated in Fig. 26. Results of the modeled beams are presented in Tables 12 and 13 and Fig. 27.

**Table 12:** Ultimate torque of the modeled beams.

Beam	$T_{u}^{FE}$, kN-m
B2	73.82
B20	75.58
B21	82.50
B3	61.21
B22	63.66
B23	70.875

**Table 13:** Results of the modeled beams.

Beam	$T_{u}^{FE}$, kN-m	$\theta_{ult}^{FE}$
B11	77.65	0.0122
B12	65.11	0.0169
B13	54.84	0.0183
B14	64.09	0.0119
B15	54.72	0.0187
B16	46.93	0.0225
B17	52.56	0.0118
B18	42.84	0.0197
B19	34.49	0.0235

Figure 27 confirmed the same trend observed in Fig. 25, highlighting that the height of the central opening had a significant impact on the maximum strain value of *UHPC* beams. Beam *B*11 exhibited higher maximum concrete strain value compared to Beam *B*14, despite the latter having a larger opening height. Similarly, Beam *B*14 recorded higher max concrete strain value than Beam *B*17, which had an even greater opening height than *B*14. This trend was consistently observed across the other beam sets: *B*12, *B*15, and *B*18, as well as *B*13, *B*16, and *B*19, where beams with smaller opening height showed higher max strain value than those with larger openings.

With respect to the ultimate angle of twist ${\varvec{\theta}}_{{{\varvec{ult}}}}^{{{\varvec{FE}}}}$, the behavior of the Beams (*B*2 to *B*10) in Table 11 closely resembled that of the Beams (*B*11 to *B*19) in Table 13. This consistency confirmed the noticeable effect of central opening length on ${\varvec{\theta}}_{{{\varvec{ult}}}}^{{{\varvec{FE}}}}$, whereas the effect of its height on ${\varvec{\theta}}_{{{\varvec{ult}}}}^{{{\varvec{FE}}}}$ appeared to be less pronounced.

The results confirmed that increasing the compression pre-force from 150 to 300kN did not affect the overall behavior of the modeled *UHPC* beams.

Table 12 illustrates that the closer the openings were positioned horizontally to the beam center, the lower the ultimate torsional strength ${\varvec{T}}_{{\varvec{u}}}^{{{\varvec{FE}}}}$. Specifically, ${\varvec{T}}_{{\varvec{u}}}^{{{\varvec{FE}}}}$ of Beam *B*21 was higher than that of Beam *B*20, which in turn was greater than that of Beam *B*2, although the three beams had the same total opening size. A similar trend was observed for Beams *B*23, *B*22 and *B*3, respectively.

## Conclusions

The effect of central openings on the torsional behavior of *UHPC* pre-compressed hollow beams was experimentally and numerically investigated in this study. It is recommended to adopt the smallest possible central opening size in *UHPC* beams as:

1. Introducing a central opening in *UHPC* simple beam with length and height ratios of 0.20 and 0.30, respectively, decreased the ultimate and cracking torque by 20% and 7%, respectively. This reduction increased to 27% and 48%, when central opening height and length ratios increased to 0.50 and 0.60, respectively.
2. The elastic stiffness of *UHPC* beam with small central opening size (length and height ratios of 0.20 and 0.30, respectively), decreased by 70% compared to the reference *UHPC* beam without opening.
3. Introducing a central opening with length to 0.40 of beam length decreased the plastic stiffness by 59% in comparison with beam without an opening.
4. The torsional ductility of *UHPC* beam with small central opening size (length and height ratios of 0.20 and 0.30, respectively), was decreased by 42% when compared to the reference *UHPC* beam with no openings.
5. The more the central opening size, the less the post-cracking load-carrying capacity of the tested *UHPC* beams.
6. The maximum concrete strain value of the reference beam was almost twice that of the beam with the smallest central opening size.
7. A direct proportionality was emerged between central opening dimensions and the values of strain in lower reinforcing steel bars, at the same loading stage.
8. Increasing the central opening height led to a great reduction in the maximum concrete strain of each tested *UHPC* beam.

Despite the drawbacks associated with the presence of openings, their inclusion also contributed to:

9. A larger central opening was associated with improved torsional ductility in the tested *UHPC* beams.
10. Increasing the central opening length did not lead to an increase in the maximum strain of the tested *UHPC* beams, having the same central opening height.
11. The tested *UHPC* beams with the same central opening height and different length showed approximately similar values of maximum concrete strain, illustrating the minor effect of changing the central opening length.

Finally, its recommended to:

12. Position web openings as far as possible from the mid-span of *UHPC* beams, as the farther the openings are located from the beam center, the lesser their effect on the beam torsional strength.

## Data Availability

The data that support the findings of this study are available from the corresponding author upon reasonable request.
